# Supplementary material for: Modeling glioblastoma heterogeneity as a dynamic network of cell states
Source: Mol Syst Biol. 2021 Sep 16;17(9):e10105. doi: 10.15252/msb.202010105 (PMC8444284; doi:10.15252/msb.202010105)
Supplement: Supplementary file 5 — Source Data for Figure 3 [file MSB-17-e10105-s001.zip › Figure3A_sourcedata/GSEA_3065/nosigdb_state3.GseaPreranked.1622718426028/FELDMAN_2020_NEURAL G0.html]

Details for gene set FELDMAN\_2020\_NEURAL G0[GSEA]

|  || Dataset | state3 |
| Phenotype | NoPhenotypeAvailable |
| Upregulated in class | na\_neg |
| GeneSet | FELDMAN\_2020\_NEURAL G0 |
| Enrichment Score (ES) | -0.38939932 |
| Normalized Enrichment Score (NES) | -1.6007092 |
| Nominal p-value | 0.0 |
| FDR q-value | 0.031800963 |
| FWER p-Value | 0.481 |
Table: GSEA Results Summary

  

Fig 1: Enrichment plot: FELDMAN\_2020\_NEURAL G0      
 Profile of the Running ES Score & Positions of GeneSet Members on the Rank Ordered List

  

| PROBE | GENE SYMBOL | GENE\_TITLE | RANK IN GENE LIST | RANK METRIC SCORE | RUNNING ES | CORE ENRICHMENT || 1 | SCRG1 |  |  | 3 | 0.645 | 0.0087 | No |
| 2 | CENPF |  |  | 4 | 0.619 | 0.0174 | No |
| 3 | PRKDC |  |  | 6 | 0.588 | 0.0255 | No |
| 4 | HSP90B1 |  |  | 7 | 0.563 | 0.0334 | No |
| 5 | PTPRZ1 |  |  | 14 | 0.532 | 0.0402 | No |
| 6 | PLAT |  |  | 18 | 0.513 | 0.0470 | No |
| 7 | PTN |  |  | 22 | 0.493 | 0.0536 | No |
| 8 | ASPM |  |  | 25 | 0.480 | 0.0601 | No |
| 9 | GPM6A |  |  | 37 | 0.460 | 0.0653 | No |
| 10 | SQLE |  |  | 39 | 0.457 | 0.0716 | No |
| 11 | TOP2A |  |  | 42 | 0.448 | 0.0776 | No |
| 12 | GGH |  |  | 46 | 0.439 | 0.0834 | No |
| 13 | FGFBP3 |  |  | 51 | 0.431 | 0.0890 | No |
| 14 | MALAT1 |  |  | 57 | 0.412 | 0.0942 | No |
| 15 | MKI67 |  |  | 59 | 0.407 | 0.0998 | No |
| 16 | HSP90AA1 |  |  | 60 | 0.402 | 0.1054 | No |
| 17 | PON2 |  |  | 63 | 0.389 | 0.1107 | No |
| 18 | HSP90AB1 |  |  | 74 | 0.371 | 0.1147 | No |
| 19 | ITM2B |  |  | 82 | 0.361 | 0.1189 | No |
| 20 | SLC44A1 |  |  | 83 | 0.361 | 0.1240 | No |
| 21 | SOX6 |  |  | 88 | 0.358 | 0.1285 | No |
| 22 | GPM6B |  |  | 105 | 0.343 | 0.1314 | No |
| 23 | MFGE8 |  |  | 107 | 0.341 | 0.1361 | No |
| 24 | SYT11 |  |  | 111 | 0.335 | 0.1404 | No |
| 25 | SMC2 |  |  | 125 | 0.322 | 0.1434 | No |
| 26 | CENPE |  |  | 126 | 0.321 | 0.1479 | No |
| 27 | SCARB2 |  |  | 132 | 0.318 | 0.1518 | No |
| 28 | SGCB |  |  | 133 | 0.317 | 0.1562 | No |
| 29 | BCHE |  |  | 136 | 0.315 | 0.1604 | No |
| 30 | DNMT1 |  |  | 149 | 0.310 | 0.1633 | No |
| 31 | LAMP2 |  |  | 154 | 0.309 | 0.1672 | No |
| 32 | KPNA2 |  |  | 157 | 0.308 | 0.1713 | No |
| 33 | PRCP |  |  | 166 | 0.303 | 0.1746 | No |
| 34 | TTYH1 |  |  | 183 | 0.293 | 0.1768 | No |
| 35 | DLGAP5 |  |  | 207 | 0.279 | 0.1780 | No |
| 36 | LAPTM4A |  |  | 208 | 0.278 | 0.1819 | No |
| 37 | DNAJC1 |  |  | 227 | 0.272 | 0.1835 | No |
| 38 | DCBLD2 |  |  | 243 | 0.265 | 0.1855 | No |
| 39 | DHCR24 |  |  | 244 | 0.265 | 0.1892 | No |
| 40 | ARL6IP5 |  |  | 245 | 0.265 | 0.1929 | No |
| 41 | SSR3 |  |  | 264 | 0.255 | 0.1944 | No |
| 42 | SMC4 |  |  | 265 | 0.255 | 0.1979 | No |
| 43 | TMEM59 |  |  | 269 | 0.253 | 0.2011 | No |
| 44 | NOVA1 |  |  | 270 | 0.253 | 0.2047 | No |
| 45 | COL9A3 |  |  | 278 | 0.250 | 0.2074 | No |
| 46 | ANLN |  |  | 281 | 0.249 | 0.2106 | No |
| 47 | CDH2 |  |  | 283 | 0.249 | 0.2140 | No |
| 48 | ASAH1 |  |  | 295 | 0.244 | 0.2161 | No |
| 49 | CADM4 |  |  | 299 | 0.244 | 0.2192 | No |
| 50 | EDNRB |  |  | 305 | 0.242 | 0.2220 | No |
| 51 | SERPINE2 |  |  | 308 | 0.241 | 0.2251 | No |
| 52 | DHRS7 |  |  | 323 | 0.238 | 0.2268 | No |
| 53 | ECE1 |  |  | 330 | 0.234 | 0.2294 | No |
| 54 | MAP2 |  |  | 344 | 0.231 | 0.2311 | No |
| 55 | SPARC |  |  | 361 | 0.228 | 0.2324 | No |
| 56 | KIF20B |  |  | 411 | 0.216 | 0.2296 | No |
| 57 | TMPO |  |  | 420 | 0.214 | 0.2316 | No |
| 58 | WSB1 |  |  | 435 | 0.212 | 0.2329 | No |
| 59 | MAGED2 |  |  | 444 | 0.210 | 0.2349 | No |
| 60 | NDFIP1 |  |  | 464 | 0.206 | 0.2356 | No |
| 61 | LAMA4 |  |  | 496 | 0.199 | 0.2347 | No |
| 62 | LRRC4C |  |  | 509 | 0.197 | 0.2360 | No |
| 63 | NT5E |  |  | 511 | 0.197 | 0.2386 | No |
| 64 | FDPS |  |  | 518 | 0.196 | 0.2407 | No |
| 65 | RRM1 |  |  | 537 | 0.194 | 0.2413 | No |
| 66 | TPX2 |  |  | 544 | 0.193 | 0.2433 | No |
| 67 | LRRN3 |  |  | 574 | 0.189 | 0.2425 | No |
| 68 | EFHC1 |  |  | 581 | 0.189 | 0.2444 | No |
| 69 | SCD5 |  |  | 619 | 0.184 | 0.2426 | No |
| 70 | FLNA |  |  | 633 | 0.181 | 0.2436 | No |
| 71 | RNF13 |  |  | 635 | 0.181 | 0.2460 | No |
| 72 | AKAP12 |  |  | 640 | 0.180 | 0.2480 | No |
| 73 | NUF2 |  |  | 680 | 0.174 | 0.2458 | No |
| 74 | PLOD2 |  |  | 683 | 0.173 | 0.2480 | No |
| 75 | COL11A1 |  |  | 684 | 0.173 | 0.2505 | No |
| 76 | MYH9 |  |  | 688 | 0.173 | 0.2525 | No |
| 77 | BAMBI |  |  | 701 | 0.170 | 0.2535 | No |
| 78 | CCT5 |  |  | 746 | 0.165 | 0.2505 | No |
| 79 | CST3 |  |  | 749 | 0.164 | 0.2526 | No |
| 80 | EZR |  |  | 759 | 0.162 | 0.2538 | No |
| 81 | CLDND1 |  |  | 773 | 0.160 | 0.2545 | No |
| 82 | SCG2 |  |  | 778 | 0.160 | 0.2563 | No |
| 83 | SDF2L1 |  |  | 801 | 0.157 | 0.2559 | No |
| 84 | MAD2L1 |  |  | 846 | 0.153 | 0.2528 | No |
| 85 | LIMCH1 |  |  | 849 | 0.153 | 0.2547 | No |
| 86 | GTSE1 |  |  | 852 | 0.152 | 0.2566 | No |
| 87 | HNRNPA2B1 |  |  | 863 | 0.151 | 0.2575 | No |
| 88 | CYR61 |  |  | 873 | 0.150 | 0.2586 | No |
| 89 | EBP |  |  | 897 | 0.149 | 0.2579 | No |
| 90 | TUBB2B |  |  | 904 | 0.148 | 0.2593 | No |
| 91 | CDK1 |  |  | 915 | 0.147 | 0.2601 | No |
| 92 | CLSPN |  |  | 922 | 0.146 | 0.2615 | No |
| 93 | MCM7 |  |  | 949 | 0.143 | 0.2604 | No |
| 94 | CLU |  |  | 988 | 0.139 | 0.2578 | No |
| 95 | ORC6 |  |  | 1000 | 0.138 | 0.2584 | No |
| 96 | LMNB1 |  |  | 1021 | 0.137 | 0.2580 | No |
| 97 | TPST2 |  |  | 1028 | 0.136 | 0.2592 | No |
| 98 | ACTN1 |  |  | 1079 | 0.132 | 0.2551 | No |
| 99 | IFI6 |  |  | 1089 | 0.131 | 0.2558 | No |
| 100 | CCNB1 |  |  | 1152 | 0.126 | 0.2502 | No |
| 101 | LINC00461 |  |  | 1272 | 0.117 | 0.2377 | No |
| 102 | TXNIP |  |  | 1317 | 0.114 | 0.2340 | No |
| 103 | SRSF7 |  |  | 1328 | 0.113 | 0.2344 | No |
| 104 | NUSAP1 |  |  | 1338 | 0.113 | 0.2349 | No |
| 105 | SPP1 |  |  | 1358 | 0.111 | 0.2342 | No |
| 106 | ESCO2 |  |  | 1373 | 0.110 | 0.2341 | No |
| 107 | FCGRT |  |  | 1410 | 0.108 | 0.2313 | No |
| 108 | TSPAN6 |  |  | 1430 | 0.107 | 0.2305 | No |
| 109 | TMEM237 |  |  | 1453 | 0.105 | 0.2294 | No |
| 110 | SPRY1 |  |  | 1471 | 0.105 | 0.2288 | No |
| 111 | PCSK1N |  |  | 1484 | 0.104 | 0.2289 | No |
| 112 | PBK |  |  | 1524 | 0.102 | 0.2257 | No |
| 113 | TSPAN9 |  |  | 1577 | 0.099 | 0.2208 | No |
| 114 | FAM181B |  |  | 1629 | 0.096 | 0.2161 | No |
| 115 | SRSF2 |  |  | 1697 | 0.092 | 0.2094 | No |
| 116 | ZKSCAN1 |  |  | 1711 | 0.091 | 0.2091 | No |
| 117 | OLIG2 |  |  | 1742 | 0.089 | 0.2068 | No |
| 118 | AURKB |  |  | 1838 | 0.084 | 0.1967 | No |
| 119 | PRSS23 |  |  | 1890 | 0.081 | 0.1917 | No |
| 120 | CNPY2 |  |  | 1924 | 0.080 | 0.1889 | No |
| 121 | EXOSC8 |  |  | 1955 | 0.078 | 0.1864 | No |
| 122 | PSAP |  |  | 1988 | 0.076 | 0.1837 | No |
| 123 | PLP1 |  |  | 2047 | 0.074 | 0.1778 | No |
| 124 | CDCA3 |  |  | 2232 | 0.067 | 0.1568 | No |
| 125 | DEPDC1 |  |  | 2259 | 0.066 | 0.1547 | No |
| 126 | IDI1 |  |  | 2285 | 0.065 | 0.1526 | No |
| 127 | EIF3E |  |  | 2496 | 0.057 | 0.1284 | No |
| 128 | DLG1 |  |  | 2526 | 0.056 | 0.1257 | No |
| 129 | CCNA2 |  |  | 2529 | 0.056 | 0.1262 | No |
| 130 | UBE2T |  |  | 2599 | 0.054 | 0.1188 | No |
| 131 | ARMCX3 |  |  | 2665 | 0.051 | 0.1117 | No |
| 132 | HSPD1 |  |  | 2700 | 0.050 | 0.1084 | No |
| 133 | ACAT2 |  |  | 2779 | 0.048 | 0.0998 | No |
| 134 | RRM2 |  |  | 2835 | 0.046 | 0.0938 | No |
| 135 | VRK1 |  |  | 2899 | 0.044 | 0.0870 | No |
| 136 | KNSTRN |  |  | 2976 | 0.042 | 0.0785 | No |
| 137 | INSIG1 |  |  | 3136 | 0.038 | 0.0601 | No |
| 138 | KIF4A |  |  | 3195 | 0.036 | 0.0536 | No |
| 139 | CKAP2 |  |  | 3236 | 0.035 | 0.0494 | No |
| 140 | SRSF3 |  |  | 3283 | 0.034 | 0.0444 | No |
| 141 | IFITM3 |  |  | 3449 | 0.029 | 0.0251 | No |
| 142 | CCNB2 |  |  | 3502 | 0.028 | 0.0193 | No |
| 143 | AURKA |  |  | 3503 | 0.028 | 0.0197 | No |
| 144 | NUDT4 |  |  | 3513 | 0.028 | 0.0190 | No |
| 145 | SRPX |  |  | 3518 | 0.028 | 0.0189 | No |
| 146 | IGFBP5 |  |  | 3555 | 0.027 | 0.0150 | No |
| 147 | SPC25 |  |  | 3575 | 0.026 | 0.0131 | No |
| 148 | PDHX |  |  | 3853 | 0.019 | -0.0197 | No |
| 149 | PRC1 |  |  | 3878 | 0.018 | -0.0223 | No |
| 150 | PLD3 |  |  | 3894 | 0.018 | -0.0238 | No |
| 151 | SNRPA1 |  |  | 3957 | 0.016 | -0.0310 | No |
| 152 | PCNA |  |  | 3986 | 0.016 | -0.0341 | No |
| 153 | CTSV |  |  | 4007 | 0.015 | -0.0363 | No |
| 154 | LOXL1 |  |  | 4025 | 0.015 | -0.0381 | No |
| 155 | PFKP |  |  | 4040 | 0.014 | -0.0396 | No |
| 156 | PLAUR |  |  | 4075 | 0.013 | -0.0434 | No |
| 157 | TROAP |  |  | 4205 | 0.009 | -0.0587 | No |
| 158 | CEP55 |  |  | 4234 | 0.009 | -0.0619 | No |
| 159 | PPA1 |  |  | 4255 | 0.008 | -0.0642 | No |
| 160 | SAT2 |  |  | 4311 | 0.006 | -0.0706 | No |
| 161 | PA2G4 |  |  | 4363 | 0.005 | -0.0766 | No |
| 162 | HMGB3 |  |  | 4445 | 0.003 | -0.0863 | No |
| 163 | SOX9 |  |  | 4503 | 0.001 | -0.0930 | No |
| 164 | TIMP4 |  |  | 4531 | 0.000 | -0.0962 | No |
| 165 | PLP2 |  |  | 4543 | 0.000 | -0.0976 | No |
| 166 | TMEFF2 |  |  | 4708 | -0.004 | -0.1170 | No |
| 167 | MIS18A |  |  | 4728 | -0.004 | -0.1193 | No |
| 168 | PAPSS1 |  |  | 4918 | -0.009 | -0.1417 | No |
| 169 | ZWINT |  |  | 4930 | -0.009 | -0.1428 | No |
| 170 | RMI2 |  |  | 5088 | -0.013 | -0.1614 | No |
| 171 | CA2 |  |  | 5274 | -0.018 | -0.1832 | No |
| 172 | PLAU |  |  | 5363 | -0.020 | -0.1934 | No |
| 173 | HMGB2 |  |  | 5479 | -0.023 | -0.2068 | No |
| 174 | CENPA |  |  | 5506 | -0.024 | -0.2095 | No |
| 175 | FRMD4A |  |  | 5530 | -0.025 | -0.2119 | No |
| 176 | IGFBP7 |  |  | 5667 | -0.029 | -0.2277 | No |
| 177 | TPM1 |  |  | 5672 | -0.029 | -0.2278 | No |
| 178 | MOXD1 |  |  | 5709 | -0.030 | -0.2317 | No |
| 179 | CDC20 |  |  | 5818 | -0.033 | -0.2441 | No |
| 180 | CENPM |  |  | 5855 | -0.034 | -0.2479 | No |
| 181 | CCDC34 |  |  | 5889 | -0.035 | -0.2513 | No |
| 182 | CNTN1 |  |  | 5924 | -0.037 | -0.2548 | No |
| 183 | MORC4 |  |  | 5929 | -0.037 | -0.2548 | No |
| 184 | USP1 |  |  | 5963 | -0.038 | -0.2582 | No |
| 185 | SESN3 |  |  | 5988 | -0.039 | -0.2605 | No |
| 186 | TCEAL8 |  |  | 5997 | -0.040 | -0.2609 | No |
| 187 | VGLL4 |  |  | 6048 | -0.041 | -0.2663 | No |
| 188 | DKK1 |  |  | 6057 | -0.041 | -0.2667 | No |
| 189 | TUBB4B |  |  | 6059 | -0.042 | -0.2662 | No |
| 190 | MDK |  |  | 6242 | -0.048 | -0.2872 | No |
| 191 | SHISA4 |  |  | 6258 | -0.049 | -0.2883 | No |
| 192 | GMNN |  |  | 6392 | -0.052 | -0.3035 | No |
| 193 | H2AFX |  |  | 6437 | -0.054 | -0.3079 | No |
| 194 | ALDOA |  |  | 6448 | -0.054 | -0.3084 | No |
| 195 | EIF4A2 |  |  | 6481 | -0.056 | -0.3114 | No |
| 196 | QKI |  |  | 6517 | -0.057 | -0.3148 | No |
| 197 | CKS2 |  |  | 6569 | -0.059 | -0.3200 | No |
| 198 | ARHGAP29 |  |  | 6596 | -0.060 | -0.3223 | No |
| 199 | FDFT1 |  |  | 6637 | -0.063 | -0.3262 | No |
| 200 | SLC9A3R2 |  |  | 6666 | -0.064 | -0.3286 | No |
| 201 | TAGLN |  |  | 6722 | -0.067 | -0.3342 | No |
| 202 | ARL5A |  |  | 6738 | -0.068 | -0.3350 | No |
| 203 | CENPW |  |  | 6750 | -0.068 | -0.3354 | No |
| 204 | CYB5B |  |  | 6751 | -0.068 | -0.3344 | No |
| 205 | VOPP1 |  |  | 6787 | -0.070 | -0.3376 | No |
| 206 | RPL22L1 |  |  | 6882 | -0.075 | -0.3478 | No |
| 207 | C1orf21 |  |  | 6895 | -0.076 | -0.3481 | No |
| 208 | CAP1 |  |  | 6922 | -0.077 | -0.3502 | No |
| 209 | HEY1 |  |  | 6933 | -0.077 | -0.3503 | No |
| 210 | DNAJC9 |  |  | 6958 | -0.078 | -0.3520 | No |
| 211 | C4orf48 |  |  | 6973 | -0.079 | -0.3526 | No |
| 212 | DECR1 |  |  | 6981 | -0.079 | -0.3523 | No |
| 213 | CLIC1 |  |  | 7041 | -0.084 | -0.3582 | No |
| 214 | ATP6V0E1 |  |  | 7106 | -0.088 | -0.3646 | No |
| 215 | NUDT1 |  |  | 7124 | -0.089 | -0.3653 | No |
| 216 | FHL2 |  |  | 7163 | -0.091 | -0.3686 | No |
| 217 | HMGB1 |  |  | 7231 | -0.096 | -0.3752 | No |
| 218 | BEX4 |  |  | 7241 | -0.096 | -0.3750 | No |
| 219 | ENO1 |  |  | 7257 | -0.097 | -0.3754 | No |
| 220 | PLEKHB1 |  |  | 7359 | -0.105 | -0.3859 | No |
| 221 | PSME2 |  |  | 7389 | -0.107 | -0.3879 | Yes |
| 222 | CENPV |  |  | 7393 | -0.108 | -0.3867 | Yes |
| 223 | TUBB6 |  |  | 7395 | -0.108 | -0.3853 | Yes |
| 224 | LMNA |  |  | 7412 | -0.109 | -0.3857 | Yes |
| 225 | RND2 |  |  | 7413 | -0.109 | -0.3842 | Yes |
| 226 | EIF1B |  |  | 7425 | -0.110 | -0.3839 | Yes |
| 227 | SH3BP5 |  |  | 7436 | -0.111 | -0.3836 | Yes |
| 228 | CCND1 |  |  | 7456 | -0.113 | -0.3843 | Yes |
| 229 | SNRPD1 |  |  | 7463 | -0.113 | -0.3834 | Yes |
| 230 | CITED1 |  |  | 7470 | -0.114 | -0.3825 | Yes |
| 231 | NMB |  |  | 7495 | -0.116 | -0.3837 | Yes |
| 232 | APIP |  |  | 7524 | -0.119 | -0.3854 | Yes |
| 233 | MRPL52 |  |  | 7540 | -0.122 | -0.3855 | Yes |
| 234 | GINS2 |  |  | 7545 | -0.122 | -0.3842 | Yes |
| 235 | TSC22D1 |  |  | 7546 | -0.122 | -0.3825 | Yes |
| 236 | RAB13 |  |  | 7553 | -0.122 | -0.3815 | Yes |
| 237 | MMAB |  |  | 7557 | -0.123 | -0.3802 | Yes |
| 238 | TK1 |  |  | 7568 | -0.124 | -0.3796 | Yes |
| 239 | CKS1B |  |  | 7569 | -0.124 | -0.3779 | Yes |
| 240 | SLC25A5 |  |  | 7582 | -0.126 | -0.3775 | Yes |
| 241 | DHFR |  |  | 7593 | -0.127 | -0.3770 | Yes |
| 242 | RAB9A |  |  | 7606 | -0.128 | -0.3766 | Yes |
| 243 | DUT |  |  | 7608 | -0.128 | -0.3749 | Yes |
| 244 | YPEL5 |  |  | 7616 | -0.129 | -0.3739 | Yes |
| 245 | NDUFA12 |  |  | 7620 | -0.129 | -0.3725 | Yes |
| 246 | SNRPB |  |  | 7671 | -0.136 | -0.3765 | Yes |
| 247 | LINC00511 |  |  | 7676 | -0.136 | -0.3751 | Yes |
| 248 | HPRT1 |  |  | 7679 | -0.137 | -0.3734 | Yes |
| 249 | EIF1AX |  |  | 7689 | -0.138 | -0.3725 | Yes |
| 250 | CACYBP |  |  | 7708 | -0.141 | -0.3727 | Yes |
| 251 | BIRC5 |  |  | 7735 | -0.144 | -0.3738 | Yes |
| 252 | MRPL15 |  |  | 7750 | -0.146 | -0.3734 | Yes |
| 253 | CIRBP |  |  | 7753 | -0.146 | -0.3716 | Yes |
| 254 | CENPN |  |  | 7756 | -0.147 | -0.3698 | Yes |
| 255 | DTYMK |  |  | 7757 | -0.147 | -0.3677 | Yes |
| 256 | MGLL |  |  | 7773 | -0.148 | -0.3674 | Yes |
| 257 | SMIM14 |  |  | 7774 | -0.148 | -0.3653 | Yes |
| 258 | CENPU |  |  | 7781 | -0.149 | -0.3640 | Yes |
| 259 | TYMS |  |  | 7792 | -0.150 | -0.3630 | Yes |
| 260 | IGBP1 |  |  | 7796 | -0.151 | -0.3613 | Yes |
| 261 | RPAIN |  |  | 7805 | -0.153 | -0.3601 | Yes |
| 262 | STMN1 |  |  | 7808 | -0.153 | -0.3582 | Yes |
| 263 | NME1 |  |  | 7811 | -0.154 | -0.3563 | Yes |
| 264 | CARHSP1 |  |  | 7812 | -0.154 | -0.3541 | Yes |
| 265 | TUBB |  |  | 7813 | -0.154 | -0.3520 | Yes |
| 266 | RGS20 |  |  | 7818 | -0.154 | -0.3503 | Yes |
| 267 | TRAF4 |  |  | 7838 | -0.156 | -0.3503 | Yes |
| 268 | CRIPT |  |  | 7841 | -0.157 | -0.3484 | Yes |
| 269 | DCTPP1 |  |  | 7845 | -0.158 | -0.3465 | Yes |
| 270 | CNP |  |  | 7852 | -0.158 | -0.3450 | Yes |
| 271 | ACOT7 |  |  | 7857 | -0.159 | -0.3432 | Yes |
| 272 | PFDN2 |  |  | 7875 | -0.162 | -0.3430 | Yes |
| 273 | CTNNAL1 |  |  | 7886 | -0.164 | -0.3419 | Yes |
| 274 | MZT1 |  |  | 7891 | -0.164 | -0.3401 | Yes |
| 275 | FEZ1 |  |  | 7903 | -0.166 | -0.3390 | Yes |
| 276 | H2AFZ |  |  | 7924 | -0.171 | -0.3390 | Yes |
| 277 | TPM3 |  |  | 7943 | -0.175 | -0.3387 | Yes |
| 278 | TLE1 |  |  | 7945 | -0.176 | -0.3364 | Yes |
| 279 | RAN |  |  | 7979 | -0.181 | -0.3377 | Yes |
| 280 | SH3BGRL |  |  | 7984 | -0.182 | -0.3357 | Yes |
| 281 | CDKN3 |  |  | 7998 | -0.184 | -0.3346 | Yes |
| 282 | RPA3 |  |  | 8014 | -0.188 | -0.3338 | Yes |
| 283 | POPDC3 |  |  | 8033 | -0.190 | -0.3333 | Yes |
| 284 | ST13 |  |  | 8037 | -0.191 | -0.3309 | Yes |
| 285 | WDR1 |  |  | 8058 | -0.195 | -0.3306 | Yes |
| 286 | HIST1H4C |  |  | 8075 | -0.198 | -0.3297 | Yes |
| 287 | RBP1 |  |  | 8081 | -0.200 | -0.3275 | Yes |
| 288 | ZFAND6 |  |  | 8115 | -0.207 | -0.3285 | Yes |
| 289 | SOX4 |  |  | 8123 | -0.209 | -0.3264 | Yes |
| 290 | RANBP1 |  |  | 8132 | -0.212 | -0.3244 | Yes |
| 291 | PNRC1 |  |  | 8133 | -0.212 | -0.3214 | Yes |
| 292 | PMAIP1 |  |  | 8157 | -0.217 | -0.3211 | Yes |
| 293 | BTG1 |  |  | 8162 | -0.218 | -0.3185 | Yes |
| 294 | NLRP1 |  |  | 8171 | -0.220 | -0.3164 | Yes |
| 295 | ZCCHC17 |  |  | 8207 | -0.231 | -0.3173 | Yes |
| 296 | HOPX |  |  | 8211 | -0.231 | -0.3144 | Yes |
| 297 | HPCAL1 |  |  | 8217 | -0.232 | -0.3117 | Yes |
| 298 | CMC2 |  |  | 8236 | -0.236 | -0.3106 | Yes |
| 299 | ATP6V1G1 |  |  | 8284 | -0.254 | -0.3126 | Yes |
| 300 | CALM2 |  |  | 8295 | -0.257 | -0.3102 | Yes |
| 301 | TAGLN2 |  |  | 8313 | -0.261 | -0.3086 | Yes |
| 302 | EIF4EBP1 |  |  | 8316 | -0.262 | -0.3051 | Yes |
| 303 | DCXR |  |  | 8321 | -0.264 | -0.3019 | Yes |
| 304 | PTTG1 |  |  | 8366 | -0.279 | -0.3032 | Yes |
| 305 | ARPC5 |  |  | 8367 | -0.279 | -0.2993 | Yes |
| 306 | GCHFR |  |  | 8371 | -0.280 | -0.2957 | Yes |
| 307 | DEK |  |  | 8376 | -0.284 | -0.2922 | Yes |
| 308 | YWHAH |  |  | 8384 | -0.289 | -0.2890 | Yes |
| 309 | ACTG1 |  |  | 8389 | -0.289 | -0.2854 | Yes |
| 310 | CYCS |  |  | 8405 | -0.297 | -0.2830 | Yes |
| 311 | PRDX6 |  |  | 8410 | -0.300 | -0.2793 | Yes |
| 312 | SNRNP25 |  |  | 8411 | -0.300 | -0.2751 | Yes |
| 313 | MARCKSL1 |  |  | 8415 | -0.300 | -0.2712 | Yes |
| 314 | PKM |  |  | 8420 | -0.301 | -0.2675 | Yes |
| 315 | H3F3A |  |  | 8442 | -0.311 | -0.2656 | Yes |
| 316 | ACTB |  |  | 8444 | -0.312 | -0.2613 | Yes |
| 317 | COTL1 |  |  | 8449 | -0.314 | -0.2574 | Yes |
| 318 | BEX1 |  |  | 8450 | -0.314 | -0.2530 | Yes |
| 319 | LYPD1 |  |  | 8460 | -0.321 | -0.2495 | Yes |
| 320 | ZNF428 |  |  | 8462 | -0.322 | -0.2451 | Yes |
| 321 | CDC42EP3 |  |  | 8473 | -0.326 | -0.2418 | Yes |
| 322 | C4orf3 |  |  | 8475 | -0.327 | -0.2373 | Yes |
| 323 | TAGLN3 |  |  | 8479 | -0.330 | -0.2330 | Yes |
| 324 | GTF3A |  |  | 8482 | -0.331 | -0.2286 | Yes |
| 325 | MLLT11 |  |  | 8489 | -0.335 | -0.2246 | Yes |
| 326 | DRAP1 |  |  | 8498 | -0.340 | -0.2208 | Yes |
| 327 | TUBA1A |  |  | 8499 | -0.341 | -0.2160 | Yes |
| 328 | ID3 |  |  | 8502 | -0.341 | -0.2115 | Yes |
| 329 | PFN2 |  |  | 8503 | -0.342 | -0.2067 | Yes |
| 330 | SIVA1 |  |  | 8511 | -0.344 | -0.2027 | Yes |
| 331 | HMGA1 |  |  | 8519 | -0.346 | -0.1986 | Yes |
| 332 | LGALS3 |  |  | 8522 | -0.347 | -0.1940 | Yes |
| 333 | PTMA |  |  | 8523 | -0.348 | -0.1891 | Yes |
| 334 | CHCHD2 |  |  | 8525 | -0.348 | -0.1844 | Yes |
| 335 | RPS27L |  |  | 8528 | -0.351 | -0.1797 | Yes |
| 336 | SAT1 |  |  | 8533 | -0.354 | -0.1752 | Yes |
| 337 | FABP7 |  |  | 8536 | -0.354 | -0.1704 | Yes |
| 338 | C12orf75 |  |  | 8548 | -0.363 | -0.1667 | Yes |
| 339 | POLD2 |  |  | 8549 | -0.363 | -0.1615 | Yes |
| 340 | S100B |  |  | 8562 | -0.373 | -0.1577 | Yes |
| 341 | METRN |  |  | 8563 | -0.374 | -0.1525 | Yes |
| 342 | PDCD5 |  |  | 8583 | -0.387 | -0.1493 | Yes |
| 343 | TMEM158 |  |  | 8586 | -0.391 | -0.1441 | Yes |
| 344 | CSRP2 |  |  | 8589 | -0.393 | -0.1388 | Yes |
| 345 | TPM4 |  |  | 8594 | -0.396 | -0.1337 | Yes |
| 346 | ITGB1BP1 |  |  | 8599 | -0.402 | -0.1285 | Yes |
| 347 | LSM4 |  |  | 8611 | -0.413 | -0.1240 | Yes |
| 348 | HMGN2 |  |  | 8644 | -0.449 | -0.1216 | Yes |
| 349 | EEF1A1 |  |  | 8646 | -0.450 | -0.1154 | Yes |
| 350 | ARPC2 |  |  | 8647 | -0.450 | -0.1090 | Yes |
| 351 | UBE2S |  |  | 8655 | -0.458 | -0.1034 | Yes |
| 352 | TXN |  |  | 8662 | -0.467 | -0.0976 | Yes |
| 353 | PPP1R14B |  |  | 8674 | -0.484 | -0.0921 | Yes |
| 354 | MZT2B |  |  | 8681 | -0.495 | -0.0859 | Yes |
| 355 | PRELID1 |  |  | 8684 | -0.498 | -0.0791 | Yes |
| 356 | S100A16 |  |  | 8714 | -0.555 | -0.0748 | Yes |
| 357 | S100A10 |  |  | 8717 | -0.558 | -0.0672 | Yes |
| 358 | PFN1 |  |  | 8732 | -0.624 | -0.0601 | Yes |
| 359 | RPSA |  |  | 8736 | -0.657 | -0.0512 | Yes |
| 360 | TUBA1B |  |  | 8745 | -0.733 | -0.0419 | Yes |
| 361 | TMSB4X |  |  | 8748 | -0.920 | -0.0292 | Yes |
| 362 | FTH1 |  |  | 8750 | -0.965 | -0.0158 | Yes |
| 363 | FTL |  |  | 8751 | -1.124 | 0.0000 | Yes |
Table: GSEA details [plain text format]

  

Fig 2: FELDMAN\_2020\_NEURAL G0: Random ES distribution      
 Gene set null distribution of ES for **FELDMAN\_2020\_NEURAL G0**
